# Sustainable growth in cattle: a global bibliometric analysis of physiological research trends

**DOI:** 10.1016/j.jgeb.2026.100854

**Published:** 2026-11-09

**Authors:** Ni Made Ayu Gemuh Rasa Astiti, I Gusti Ayu Dewi Seri Rejeki, Ahmad Fudholi

**Affiliations:** a Department of Postgraduate Agricultural Science, Warmadewa University, Denpasar 80235, Indonesia; b Department of Animal Husbandry, Warmadewa University, Denpasar 80235, Indonesia; c National Research and Innovation Agency, South Tangerang 15314, Indonesia; d Research Collaboration Centre for Carbon Materials-based Biomass for Conversion and Energy Storage, Universitas Riau and National Research and Innovation Agency, Riau, Indonesia; e Pusat Pengajian Citra Universiti, Universiti Kebangsaan Malaysia, Bangi, Selangor, 43600, Malaysia

**Keywords:** Sustainable, Growth, Cattle, Bibliometric, Physiological

## Abstract

Understanding the sustainability of physiological growth processes in cattle requires integrating developmental biology, nutrition, genetics, and environmental factors. This study aimed to map global research trends and synthesize current insights on cattle growth physiology using a bibliometric approach to identify emerging themes and knowledge gaps. A structured bibliometric search was conducted to retrieve peer-reviewed literature on cattle growth, emphasizing prenatal and postnatal development, hormonal regulation, genetic selection, nutritional strategies, environmental stressors, and advanced analytical tools such as metabolomics and proteomics. Co-occurrence mapping and thematic clustering were applied to determine dominant research patterns and converging scientific domains. Findings indicated a clear developmental continuum from fetal programming to physiological maturity, underscoring the roles of maternal nutrition, endocrine modulation, and tissue-specific growth dynamics. Recent research has highlighted a growing focus on feed efficiency, metabolomics, and proteomics, reflecting a shift toward a molecular-level understanding of nutrient partitioning, metabolic adaptation, and growth regulation. Genetic influences, including breed differences, sex-related biological variation, and traits linked to residual feed intake, were identified as major contributors to sustainable growth. Nutritional management, backgrounding practices, and environmental stressors, particularly heat stress, significantly affected intake behavior, growth trajectories, and carcass composition. Together, these findings show that growth outcomes are shaped by interactions among genetics, early-life programming, nutrient supply, and management systems. Achieving sustainable growth in cattle requires integrated strategies that unite developmental physiology, precision nutrition, adaptive genetic selection, and resilience to environmental stress, providing a clear foundation for future research to enhance efficiency, sustainability, and animal welfare.

## 1 Introduction

Growth physiology in cattle is governed by an intricate interplay of genetic, nutritional, and environmental factors that collectively shape developmental trajectories, metabolic efficiency, and overall productivity. Central to these processes is the development of the gastrointestinal system, particularly the rumen, which undergoes profound morphological and functional changes during early life. Although calves are born functionally pseudo-monogastric, rapid ruminal development is required for a successful transition to the ruminant state, enabling efficient digestion of fibrous feeds and supporting long-term performance. Early-life nutrition strongly influences this developmental stage by modulating rumen papillae growth, microbial colonization, epithelial maturation, and fermentation patterns. As emphasized in previous reviews, strategic provisioning of solid feeds, fermentable carbohydrates, and bioactive compounds can accelerate rumen maturation, with direct consequences for nutrient absorption, feed intake capacity, and subsequent growth.^1^ Early nutritional interventions, therefore, represent a critical leverage point for optimizing the long-term utilization of dietary resources in cattle production systems.

The broader context of growth physiology extends beyond gastrointestinal development to encompass the dynamic interactions between genetics, feeding strategies, and environmental management. Advances in dairy and beef cattle nutrition have demonstrated that improvements in ration formulation, feed digestibility, and nutrient availability can significantly enhance growth rates and production efficiency.^2^ At the same time, cattle feeding behavior, including selective consumption or feed sorting, can alter actual nutrient intake relative to the formulated diet. Such behavior, which varies across breeds and dietary strategies, ultimately affects performance by modifying rumen fermentation dynamics and nutrient utilization patterns. Studies examining intake behavior in different genetic groups have shown that feed sorting can influence dry matter intake, energy balance, and growth outcomes, underscoring the need to account for behavioral responses when designing nutritional programs.^3^

Genetic influences on growth efficiency are particularly evident in the context of residual feed intake (RFI), a trait associated with reduced feed consumption at comparable production levels. Molecular and physiological studies reveal that selection for low RFI is linked to more efficient nutrient use, altered metabolic pathways, and modifications in growth-related traits.^4^ Breed-specific responses to diet further highlight the complex genetic nutrition interactions that influence cattle productivity. For example, comparative performance evaluations of Indonesian ruminant genotypes indicate that live weight gain, feed conversion, and nutrient utilization vary significantly across breeds and dietary quality, demonstrating that genetic potential can either enhance or limit the effectiveness of nutritional interventions.^5^ These findings collectively emphasize that cattle growth cannot be fully understood without considering both genetic background and dietary context.

Feed resource availability and quality also play a pivotal role in shaping growth performance, especially in systems reliant on forage-based diets. The use of alternative or supplemental feed ingredients has gained attention as a means to enhance nutrient intake and reduce feed costs. Research on rice-based feed ingredients, for instance, has demonstrated promising nutritive value for ruminants, with potential applications in cattle rations where energy density or feed availability are limiting factors.^6^ Similarly, incorporating carbohydrate vitamin mineral concentrates has been shown to improve ruminal conditions and biochemical parameters, demonstrating the value of nutrient-dense supplements in supporting rumen health and growth.^7^ More recently, novel feed resources such as Panax notoginseng residue have been evaluated for their effects on rumen microbial communities and metabolic responses, revealing potential benefits for both rumen function and overall growth performance.^8^ Such diversification of feed resources is especially relevant in regions where conventional feed ingredients are scarce or economically constrained.

Beyond nutritional factors, environmental and management conditions exert a substantial influence on cattle growth. Housing systems, pasture quality, climate-related stressors, and overall husbandry practices interact with an animal's genetic and nutritional environment. For example, studies conducted in tropical and subtropical production systems show that energy requirements for maintenance and growth can vary considerably depending on environment-specific challenges, such as heat load or forage availability.^9^ Environmental stressors can also indirectly affect growth by altering feed intake, reducing nutrient digestibility, or impairing rumen function. These findings reinforce the importance of designing integrated management systems that accommodate environmental variability while maintaining consistent nutritional support.

A comprehensive understanding of cattle growth physiology requires an appreciation of the interconnected developmental, nutritional, genetic, and environmental influences that shape performance outcomes. Early rumen development establishes the physiological foundation for efficient nutrient utilization throughout the animal's life^1^ while advances in applied nutrition continue to enhance the precision of feeding strategies aimed at promoting optimal growth.^2^ Genetic selection for traits such as residual feed intake offers opportunities to improve metabolic efficiency at the population level,^4^ and the adoption of alternative feed resources provides resilience against fluctuations in feed supply.^6^ Meanwhile, behavioral characteristics, such as feed sorting, and environmental constraints, such as pasture quality, further modulate how cattle respond to these interventions.^3^, ^9^

This study aims to systematically map and analyze global research trends on cattle growth by applying a bibliometric approach to identify how the scientific focus has evolved from traditional physiology toward sustainability-driven themes, including heat stress, emissions reduction, and adaptive management. The objective is to integrate current physiological knowledge. Additionally, the research seeks to evaluate environmental and management factors, particularly heat stress and nutritional strategies, that significantly affect intake, thermoregulation, and methane-linked energy losses in cattle. Finally, this study aims to generate forward-looking recommendations by identifying emerging research gaps and proposing integrative future directions.

## 2 Bibliometric approach

The Boolean search strategy for the topic “Physiological Processes of Growth in Cattle” was developed using key terms related to the bovine species and growth mechanisms. The main search string combined species-specific terms (“cattle,” “bovine,” “beef cattle,” or “dairy cattle”) with growth-related concepts (“growth physiology,” “physiological growth,” “tissue development,” “muscle growth,” or “skeletal growth”) and regulatory processes (“metabolism,” “endocrine regulation,” “hormonal regulation,” or “nutrient partitioning”). To refine the results, additional filters for nutrition and feed efficiency were applied, while studies involving non-bovine species, such as humans, rodents, poultry, sheep, or goats, were excluded using the NOT operator.

The inclusion criteria comprised peer-reviewed articles and reviews published in English that focused on the physiological mechanisms governing growth in beef or dairy cattle, including prenatal and postnatal stages. Eligible studies addressed the metabolic, endocrine, or nutritional regulation of muscle, bone, adipose tissue, or organ development. Exclusion criteria included studies on non-bovine species; research emphasizing management or economic aspects without physiological analysis; articles focused solely on molecular genetics without linkage to growth physiology; and non-peer-reviewed sources such as editorials or opinion papers.

Fig. 1 shows the number of documents by year in Scopus. The bibliometric trend of Scopus-indexed publications on “Physiological Processes of Growth in Cattle” from 2015 to 2026 shows moderate but steady output in the early period (2015–2019), followed by a clear acceleration after 2020. Publication numbers increased markedly in 2021 and again in 2025, each reaching 33 documents, indicating heightened research interest likely driven by advances in growth physiology, genomics, and efficiency-oriented livestock production. Although 2022 saw a temporary decline, overall output remained consistently higher than pre-2020 levels, reflecting a strengthening, more dynamic research landscape. The sharp drop in 2026 should be interpreted cautiously, as it reflects incomplete data for that year rather than a true reduction in scientific activity.

**Fig. 1:**
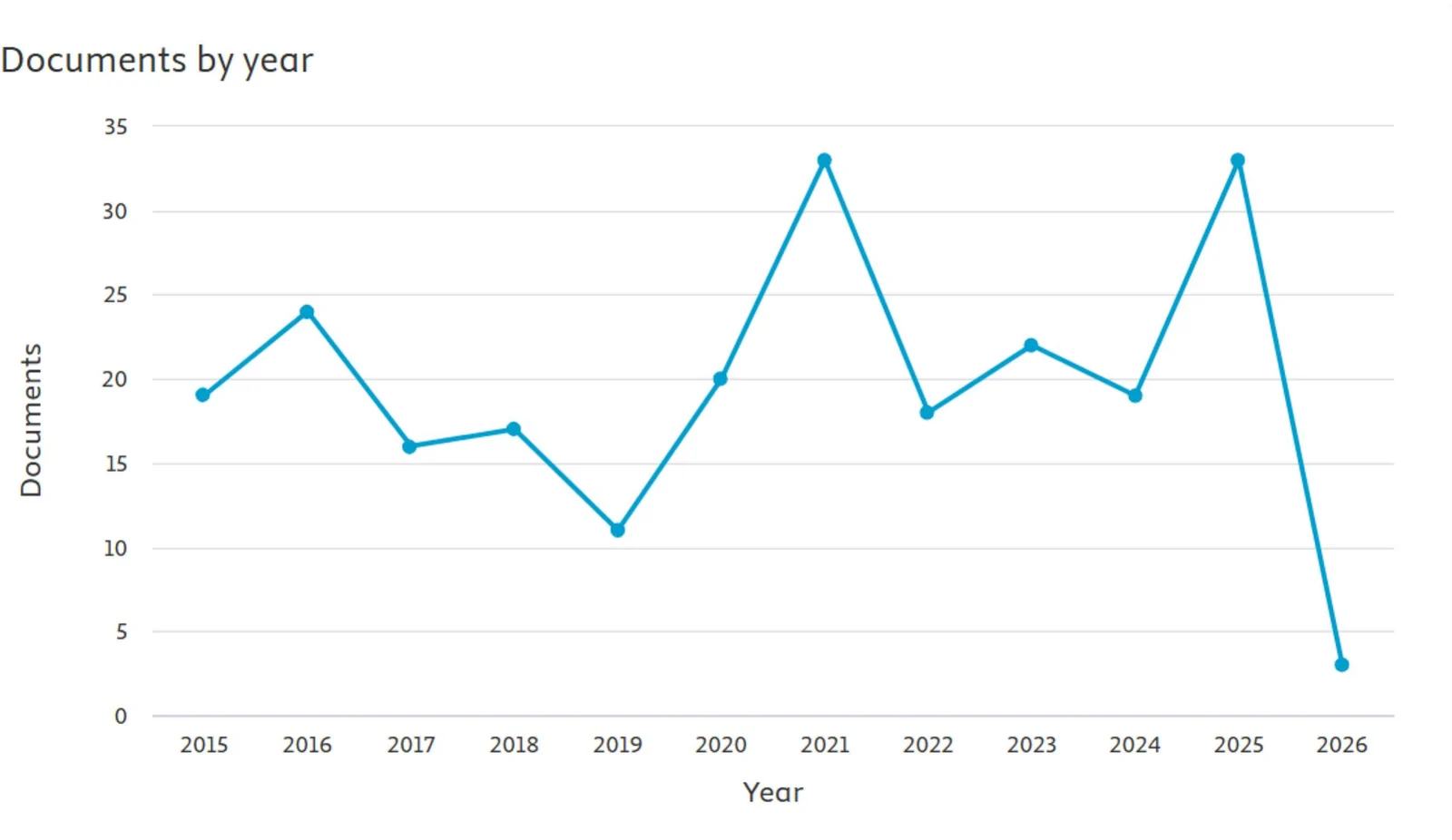
Documents by year in Scopus with keywords “Physiological Processes of Growth in Cattle”.

The surges in publications observed in 2021 and 2025 were partially attributed to the indirect effects of the COVID-19 pandemic on scientific research and agricultural systems. During the pandemic, disruptions to global food supply chains heightened concerns about food security, efficiency, and resilience in livestock production, prompting increased research attention toward cattle growth physiology, productivity, and resource optimization. In addition, lockdowns and reduced fieldwork in 2020 enabled researchers to focus more on data analysis and manuscript preparation, contributing to the spike in publications observed in 2021. The subsequent rise in 2025 likely reflects post-pandemic recovery, renewed research funding, and the accelerated adoption of advanced tools such as genomics, precision nutrition, and growth modeling, as research priorities shifted toward sustainable and efficient animal production systems informed by lessons learned during the pandemic.^10^

Fig. 2 shows the PRISMA diagram. The data were retrieved from the internationally recognized Scopus database on February 3, 2026, using the search keyword “Physiological Processes of Growth in Cattle.” The initial search yielded 470 publications published between 2015 and 2026. To ensure the relevance and quality of the dataset, a multi-stage screening procedure was implemented. During the first screening stage, the titles, abstracts, and conclusions of all retrieved records were systematically examined, resulting in the selection of 155 relevant publications. Subsequently, a more rigorous inclusion process was conducted by selecting studies that aligned with three emerging research themes identified through the overlay visualization generated using VOSviewer: feed efficiency, metabolomics, and proteomics. This final screening produced a refined dataset of 23 publications, which served as the foundation for the subsequent bibliometric and thematic analyses. The complete study selection process, together with the identification of the emerging research themes, is presented in Fig. 4 (Overlay Visualization in VOSviewer).

**Fig. 2:**
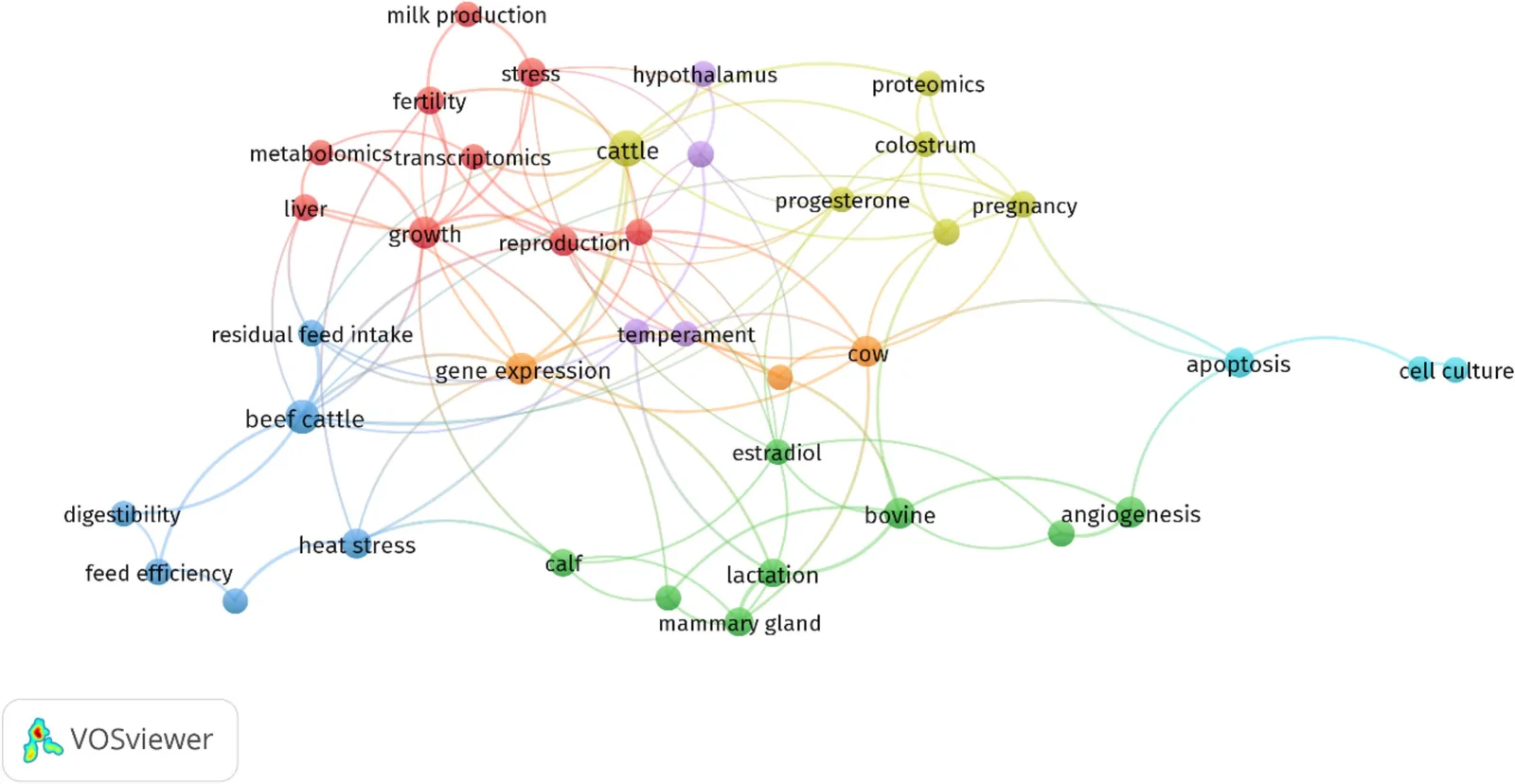
PRISMA Diagram.

Although both the network visualization and overlay visualization in VOSviewer are derived from the same keyword co-occurrence dataset, they provide complementary insights into the research field. The network visualization reveals the conceptual structure by illustrating the relationships, co-occurrence strength, and clustering of keywords, thereby identifying the major research themes and their interconnections. In contrast, the overlay visualization incorporates a temporal dimension by assigning colors to keywords based on their average publication year, enabling the identification of emerging research trends. In this study, the network visualization highlights the conceptual relationships among keywords related to the physiological processes of growth in cattle, whereas the overlay visualization identifies feed efficiency, metabolomics, and proteomics as emerging research themes, reflecting a recent shift toward molecular, metabolic, and precision-livestock approaches to understanding cattle growth. Together, these visualizations provide a comprehensive understanding of both the knowledge structure and its temporal evolution**.**

Fig. 3 shows the VOSviewer network visualization, which reveals a well-structured thematic landscape of research on the physiological processes of growth in cattle. At the core of the map, the term “cattle” acts as a central hub connecting multiple clusters, consistent with VOSviewer's method of positioning items based on similarity and co-occurrence strength. The red cluster highlights strong integration among growth, reproduction, stress, milk production, and omics-related terms, demonstrating that growth physiology is commonly studied in the context of metabolic and reproductive performance. Meanwhile, the blue cluster represents feed efficiency, residual feed intake, digestibility, and heat stress, indicating a research focus on efficiency traits and environmental adaptation, topics that co-occur due to their shared relevance in production physiology. The spatial arrangement, where closer nodes indicate stronger conceptual ties, reflects the interconnected nature of these domains.

**Fig. 3:**
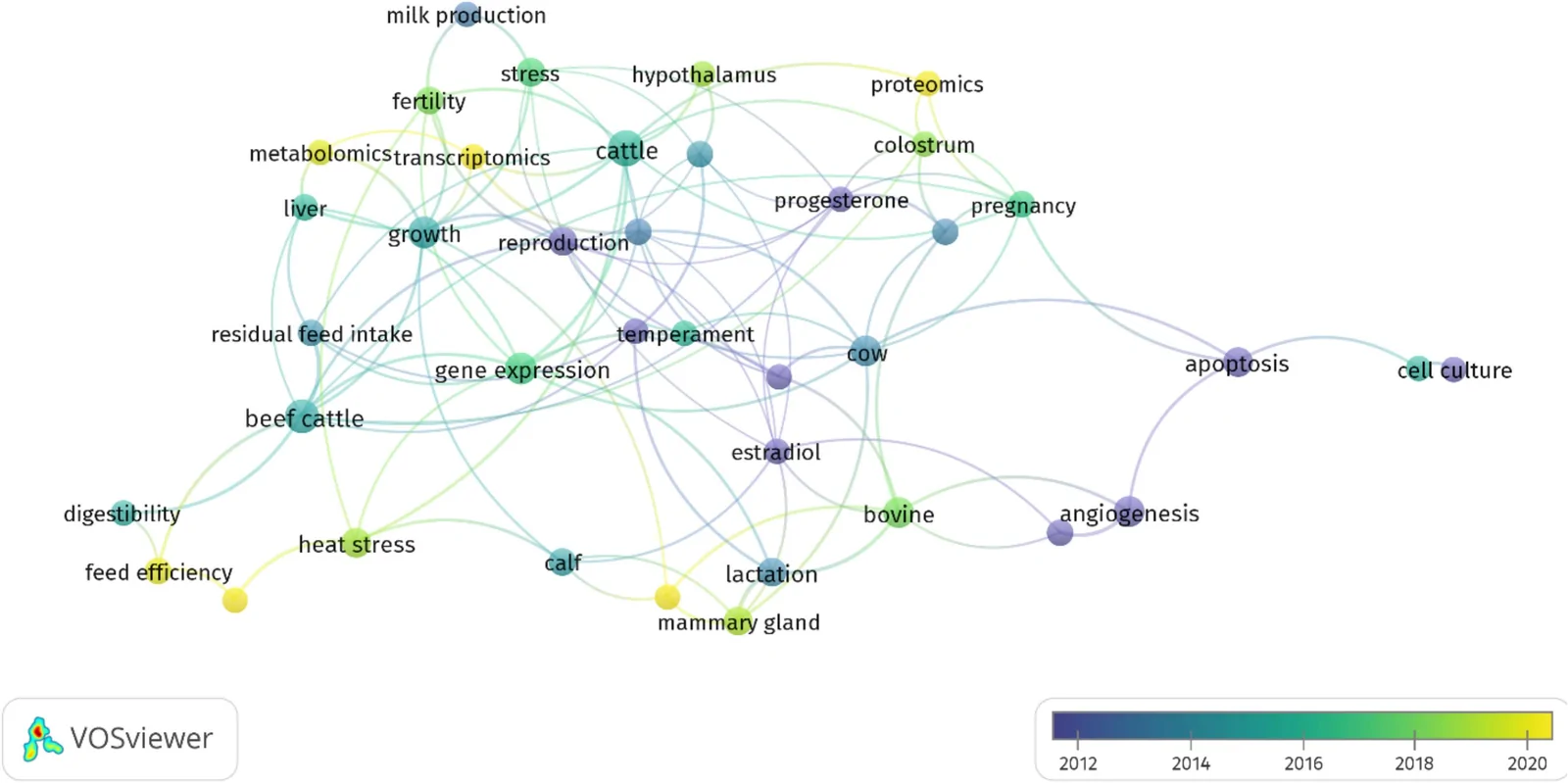
The Network Visualization in Vosviewer.

Complementing these are additional clusters that enrich the field's conceptual structure. The green cluster captures lactation, mammary gland, calf, and endocrine-linked terms such as estradiol, illustrating the importance of maternal offspring physiology in shaping growth outcomes. The yellow cluster, containing pregnancy, progesterone, colostrum, and proteomics, points toward molecular and peripartum physiological research. More specialized areas appear at the network's edges, such as the teal cluster on apoptosis and cell culture, which represents mechanistic or laboratory-based studies. According to VOSviewer principles, such peripheral positioning reflects terms with fewer, yet still meaningful, connections to the core literature. Overall, the network demonstrates that research on cattle growth physiology is inherently multidisciplinary, linking molecular biology, reproduction, nutrition, environmental stress, and developmental physiology into an integrated bibliometric ecosystem.

The VOSviewer network visualization in Fig. 3 illustrates how research themes surrounding the physiological processes of growth in cattle are intellectually organized and interconnected based on keyword co-occurrence patterns. Node size reflects the frequency of terms in the literature, while the distance and connecting lines indicate the strength of their conceptual relationships, allowing dominant and emerging themes to be easily identified. The central positioning of the term “cattle” confirms its role as the unifying research subject, linking multiple clusters that represent complementary dimensions of growth physiology. Dense linkages within the red and blue clusters indicate well-established research areas, where growth is closely associated with reproduction, metabolic regulation, milk production, feed efficiency, and adaptation to environmental stress. Surrounding clusters, such as those focused on lactation, endocrine regulation, pregnancy, and molecular approaches, add biological depth by highlighting developmental, hormonal, and omics-driven perspectives. More peripheral clusters, including cell culture and apoptosis, suggest specialized mechanistic studies that contribute foundational knowledge but are less frequently integrated into applied research. Overall, the visualization demonstrates that cattle growth physiology research is highly multidisciplinary, and VOSviewer effectively captures both the core themes and their supporting subfields within a coherent, interconnected research network.

The **first keyword** to emerge in 2020 and beyond is **feed efficiency**, as shown in Fig. 4**.** The feed efficiency of cattle is essential for sustainable beef production and has increasingly garnered research attention, particularly given the rising global demand for meat and concurrent environmental concerns. By evaluating physiological processes and genetic factors that influence growth and feed efficiency, researchers aim to enhance production efficiency while addressing environmental sustainability. This synthesis examines advances, methodologies, and the relative impact of physiological processes on cattle feed efficiency.

**Fig. 4:**
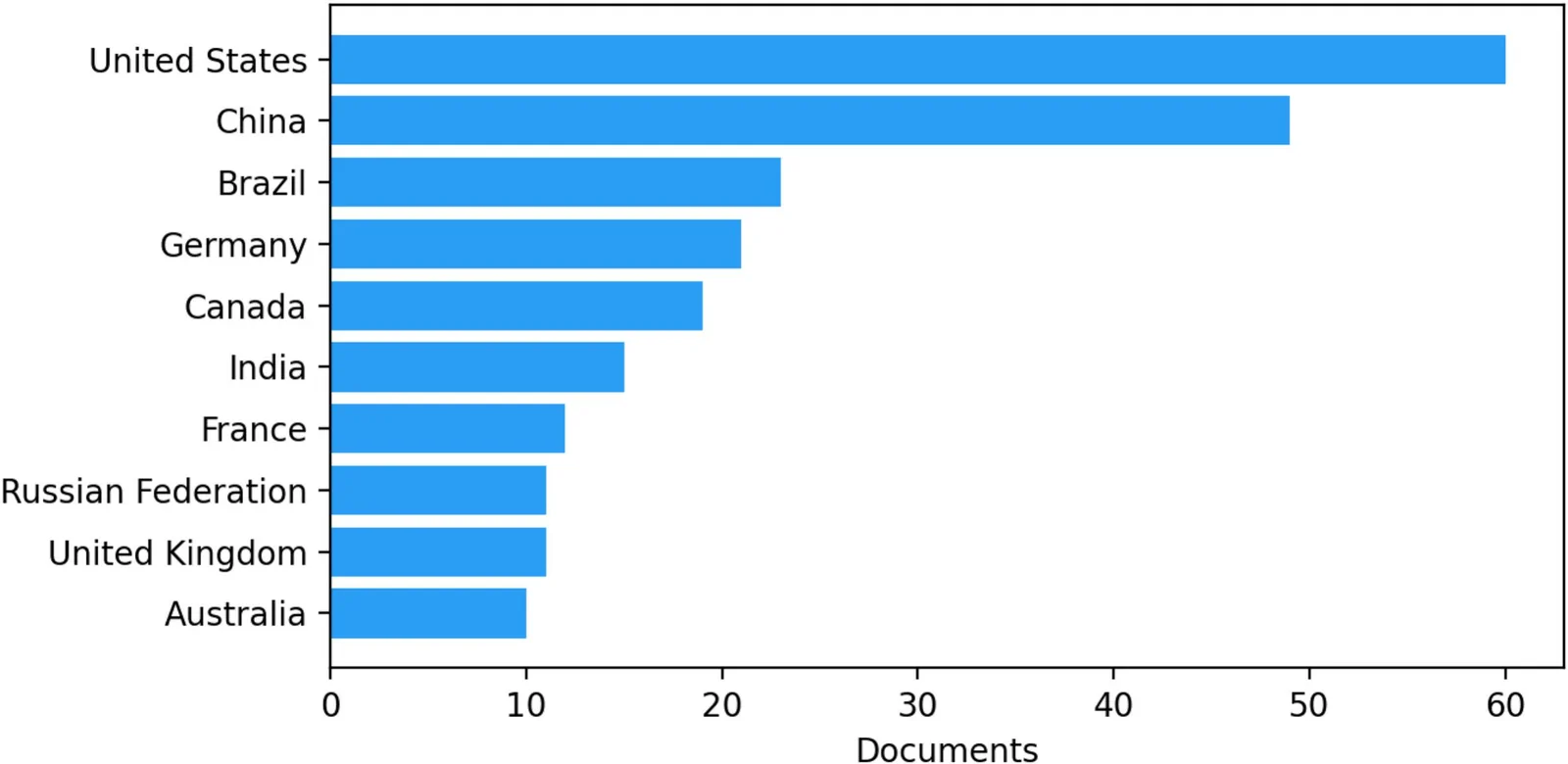
The Overlay Visualization in Vosviewer

Feed efficiency, often measured as residual feed intake (RFI) or average daily gain (ADG), is a critical factor influencing cattle productivity. Established studies indicate that physiological responses to environmental stresses, such as heat stress, play a significant role in feed efficiency. Under heat-stress conditions, cattle exhibit increased respiratory and sweating rates, leading to decreased feed intake.^11^ This impaired feed intake can adversely affect growth performance, prompting attention to metabolic adaptations that could be leveraged to improve feed efficiency. Furthermore, the liver's role in directing metabolism under heat stress suggests potential avenues for enhancing resilience and feed efficiency through nutritional modulation.^11^ Understanding these physiological processes enables the development of targeted interventions to mitigate the detrimental effects of environmental challenges on cattle production.

Significant progress has been made in identifying genes linked to feed efficiency. For instance, Abo-Ismail et al. have developed a single-nucleotide polymorphism (SNP) panel tailored for beef cattle, facilitating genetic evaluation of feed efficiency traits across populations.^12^ This SNP panel, validated in Angus and Hereford breeds, is crucial for breeding programs focused on improving feed efficiency. Additionally, extensive genome-wide association studies (GWAS) have been conducted, identifying numerous genetic markers associated with feed efficiency traits, including RFI and ADG. Seabury et al. highlighted the potential for integrating these markers into breeding programs, thereby enhancing both feed efficiency and growth traits.^13^ Identifying significant SNPs associated with RFI can lead to more precise breeding strategies that increase productivity while minimizing resource use.

Recent studies examining the effects of selection on growth phenotypes have underscored the importance of breeding practices in improving feed efficiency. Johnson et al. compared Angus-derived steer calves selected for moderate and high growth potential, observing significant differences in feed intake and performance metrics.^14^ Selection protocols that incorporate residual feed intake criteria have proven essential for improving overall feed efficiency, enabling producers to optimize growth rates relative to feed consumption. Moreover, research by Novais et al. emphasizes the need for longitudinal studies to evaluate the impacts of RFI on cattle performance in various grazing scenarios, thereby offering insights into how selection for feed efficiency traits translates into real-world settings.^15^ This study indicates that selection can lead to cattle that require less feed to achieve the same growth outcomes, enhancing economic sustainability while addressing fluctuating feed costs.

Nutritional management plays a pivotal role in achieving high feed efficiency. Recent studies indicated that optimizing dietary formulations, including the use of alternative feed ingredients and feed-grade antibiotics, can significantly improve the health and growth performance of feedlot cattle.^16^ The investigation into the impact of maternal bovine appeasing substances revealed enhancements in growth performance and feed efficiency metrics, illustrating the intricate connection between nutrition, stress management, and overall cattle productivity.^17^ Moreover, adjusting nutrient types and implementing phase feeding strategies have been shown to affect economic returns, highlighting the importance of integrating nutritional strategies with genetic improvements to maximize feed efficiency.^18^ Understanding and applying these nutritional strategies alongside genetic advancements offers a comprehensive approach to enhancing cattle feed efficiency.

The second keyword that emerged in 2020 and beyond is **metabolomics,** as shown in Fig. 4**.** Metabolomics, the comprehensive study of metabolites in biological systems, has become increasingly relevant for understanding the physiological processes of growth in cattle. This field examines the intricate biochemical pathways influenced by factors such as nutrition, stressors, and genetics, ultimately affecting growth rates, health, and productivity. Significant advancements and research findings have emerged, elucidating the metabolic underpinnings of growth in cattle.

Nutrition plays a critical role in the growth and development of cattle, influencing their metabolic profiles. Specific dietary components, such as proteins, fats, and carbohydrates, interact at the biochemical level to modulate energy balances, tissue growth, and overall health. For instance, studies have illustrated that the composition of feed, including the precise ratios of macronutrients, directly affects the metabolic pathways involved in growth and milk production.^19^, ^20^ Recent research has demonstrated a strong correlation between cattle's metabolomic profiles and their diet.^19^ Using non-targeted metabolomic profiling, researchers have identified metabolites that respond to different feed compositions, highlighting the potential to tailor nutritional strategies to optimize cattle growth.^20^ The implications of such findings include improved feed formulations that enhance metabolic efficiency, thereby promoting higher growth rates in cattle.

Cattle are often exposed to various stressors, including environmental changes, disease, and management practices, which can significantly impact their growth. Metabolomics provides a powerful tool for understanding how these stressors affect metabolic pathways. For instance, stress-induced metabolic adaptations can alter energy utilization and growth trajectories.^21^ Recent studies have focused on the impact of climate-induced stressors (like heat or cold exposure) on cattle metabolism, revealing specific biomarkers associated with stress resilience and adaptation.^22^ Furthermore, the role of gut microbiota in modulating stress responses and influencing metabolism has garnered attention. Alterations in microbiota composition seen in response to different diets or stressors are linked to fluctuations in metabolite production, which can modulate both immune responses and growth parameters.^23^, ^24^ This change highlights the interconnectedness of nutrition, microbiota, and metabolic health in promoting optimal growth conditions for cattle.

Genetic factors also contribute significantly to the differences observed in metabolic profiles among cattle breeds. Genomic studies in conjunction with metabolomics have begun to uncover how specific genetic markers influence metabolic pathways associated with growth and adaptation.^25^ Research efforts are increasingly employing multi-omics approaches that integrate genomics, transcriptomics, and metabolomics to provide a holistic view of the metabolic processes underlying growth traits in cattle.^23^ Such comprehensive methodologies enable the identification of candidate genes that can be leveraged in breeding programs to enhance growth performance and disease resistance in cattle. This genetic information, when analyzed alongside metabolomic data, reveals potential pathways that could be targeted for improving growth efficiency in livestock.^26^

The third keyword that emerged in 2020 and beyond is **proteomics,** as shown in Fig. 4. The study of proteomics in cattle physiology, particularly during growth, offers vital insights into the molecular mechanisms underlying development, health, and production traits. This review synthesizes research findings to elucidate the complex interplay among proteins involved in growth-related physiological processes in cattle. Growth hormone (GH) plays a pivotal role in stimulating growth and regulating metabolic processes in cattle. GH exerts its effects by binding to the growth hormone receptor (GHR), influencing numerous physiological traits, particularly in milk production and growth rates in beef cattle.^27^ Genetic polymorphisms within the GHR gene, such as the non-synonymous SNP F279Y, have been shown to correlate positively with growth performance traits, indicating that variations in GHR can significantly affect cattle growth and productivity outcomes.^27^

The application of proteomic technologies allows for the identification and quantification of proteins that respond dynamically to growth stimuli. For instance, studies employing proteomic profiling techniques have revealed alterations in protein expression associated with various growth conditions, including the administration of anabolic agents such as dexamethasone, which highlights how growth promoters influence plasma protein markers and overall physiological responses.^28^ Furthermore, proteomic studies targeting extracellular vesicles from bovine milk have identified proteins associated with immune responses and growth, suggesting that early biomarkers of growth may be derived from milk-derived exosomes.^29^

Post-translational modifications (PTMs) are critical in regulating protein function, particularly in response to physiological stimuli encountered during growth. Glycosylation, a common PTM, can significantly influence protein activity and signaling pathways pertinent to growth and development.^30^ However, this aspect is often under-researched in bovine proteomics, indicating a gap in current knowledge that could be pivotal in comprehending growth-related mechanisms. Advancing both global and site-specific analyses of glycosylation could enhance our understanding of these regulatory processes.^30^

Recent studies on the proteomic profiles of extracellular vesicles, particularly those derived from milk, have underscored their significance in conveying information about the host's physiological state. Proteins found in these vesicles are implicated in immunological responses essential for growth and can serve as markers for physiological changes associated with growth processes.^29^, ^31^ The characterization of these vesicles is crucial, as they may mediate cell-to-cell communication and facilitate adaptation to growth challenges, thereby influencing overall productivity and health in cattle.

As cattle grow, their proteomes exhibit significant adaptations that reflect changes in metabolic demands and environmental interactions. For example, studies have shown that increasing cell size, relevant to muscle growth, correlates with specific proteomic alterations that impact growth rates and physiological states.^32^ Coupled with the advancements in mass spectrometry and systems biology, these proteomic insights can enrich our understanding of how cattle adapt to various nutritional and environmental factors during growth.^33^

## 3 Understanding growth physiology in cattle

### 3.1 Prenatal and postnatal development

Prenatal development plays a critical role in establishing the foundational structure of muscle, adipose, and bone tissues in cattle, and these processes are best understood within clearly defined gestational stages. Bovine pregnancy is conventionally divided into three trimesters: the first trimester (conception to ~95 days of gestation), the second trimester (~96–190 days), and the third trimester (~191 days to parturition). Skeletal muscle fiber number is determined entirely before birth, with primary and secondary myogenesis occurring predominantly during the first and second trimesters (early to mid-pregnancy) under the regulation of conserved myogenic regulatory factors, including MyoD, Myf5, Myogenin, and MRF4.^34^, ^35^ Because the quantity of postnatal muscle fiber cannot be increased, nutritional, hormonal, or environmental perturbations during these early and mid-gestational windows can have lasting consequences for muscle growth potential and carcass traits. Adipogenesis follows a more staggered timeline, with adipoblasts emerging primarily during the second trimester (mid-pregnancy) and differentiating into mature adipocytes during the third trimester (late pregnancy), a phase particularly sensitive to maternal nutrition and endocrine signals that influence adipose precursor proliferation and differentiation.^36^, ^37^

This synthesis highlights the critical role of the prenatal period in establishing the foundational structure of muscle, adipose, and bone tissues in livestock. Consistent with prior reports, the number of skeletal muscle fibers was determined before birth, with primary and secondary myogenesis occurring predominantly during the first two trimesters of gestation. These developmental phases were regulated by a conserved network of myogenic regulatory factors, including MyoD, Myf5, Myogenin, and MRF4, which coordinate lineage commitment and myoblast differentiation.^34^, ^35^ Because postnatal muscle fiber number cannot be increased, any perturbations during this embryonic window, nutritional, hormonal, or environmental, may have lasting consequences for muscle growth potential and carcass traits.

Similarly, adipogenesis during gestation followed a temporally distinct pattern. Adipoblasts emerged around mid-gestation and differentiated into mature adipocytes later in fetal life. This process is sensitive to maternal nutritional status and endocrine cues, which influence the proliferation and differentiation of adipose precursors.^36^, ^37^ The PPARγ gene network, in particular, plays a central role in directing adipocyte fate and lipid-filling capacity, linking maternal nutritional inputs to long-term intramuscular adiposity outcomes.^37^ These findings underscore the importance of strategic maternal nutrition programs to optimize the balance between lean and fat deposition in offspring.

Bone mineralization also progressed early during gestation, with ossification centers forming between days 30 and 40. Mineral accretion during this phase depended heavily on maternal nutrient supply, especially calcium and phosphorus. Recent evidence demonstrated that maternal vitamin and mineral supplementation can alter fetal organ mass, endocrine status, and substrate availability as early as day 83 of gestation, further supporting the responsiveness of fetal bone and tissue development to maternal diet composition.^38^

Collectively, these results reaffirm the concept of fetal programming, in which prenatal conditions exert enduring influences on postnatal growth trajectories and tissue function. This finding is consistent with earlier ruminant developmental programming models, which emphasize the susceptibility of muscle and adipose lineages to gestational perturbations.^35^

Postnatal muscle growth occurred primarily by hypertrophy, as fiber number had already been established prenatally. Satellite cells played a pivotal role in this process by donating nuclei to growing fibers. Their activity was regulated by anabolic hormones, including growth hormone (GH), insulin-like growth factor-1 (IGF-1), and testosterone, which collectively stimulate protein synthesis and enhance myogenic capacity.^39^, ^40^ These findings suggest that both endocrine status and nutritional management during early postnatal life are central determinants of muscle mass accumulation. Bone growth continued through the action of epiphyseal plates, which remained active until puberty. The timing of epiphyseal closure is largely influenced by estrogen, which accelerates maturation and ossification, thereby dictating final skeletal dimensions.^41^ This hormonal regulation aligns with known patterns of growth plate senescence across mammalian species.

Postnatal fat deposition reflected both hypertrophy and hyperplasia, depending on the adipose depot. Visceral, subcutaneous, intermuscular, and intramuscular depots displayed distinct developmental timelines and nutrient sensitivities. High-energy diets enhanced adipocyte lipid accumulation and, in some cases, cell number expansion.^36^ Lipogenesis in bovine adipose tissue was most strongly stimulated by insulin and IGF signaling, linking metabolic status to fat accretion.^42^ These mechanisms reinforce the interplay between dietary energy supply and endocrine regulation in orchestrating patterns of fat deposition.

The combined findings from prenatal and postnatal analyses illustrate the continuity of tissue development from gestation through maturity. Prenatal gene expression patterns and maternal nutrition establish the structural template for muscle fiber number, adipocyte precursor abundance, and bone mineral reserves. Postnatal endocrine environment, nutrient intake, and physiological stimuli then determine how these tissues grow, remodel, or accumulate mass.

This developmental continuum is well captured in recent reviews emphasizing the long-term and transgenerational implications of early-life programming for livestock productivity and meat quality outcomes.^41^ The results support a growing body of evidence suggesting that optimizing maternal nutrition and early-life management practices may be among the most effective strategies for improving growth efficiency, carcass composition, and overall performance in livestock systems.

### 3.2 Effects of hormonal, age, and physiological maturity

The present synthesis demonstrates the differential and complementary roles of estrogen, progesterone, and testosterone in regulating adipose, muscle, and bone development in cattle. Estrogen was shown to promote adipogenesis, partly by enhancing insulin sensitivity and stimulating lipid-filling pathways in intramuscular adipocytes.^43^, ^44^ Additionally, estrogen accelerates bone maturation, consistent with its well-established function in driving growth plate ossification and shaping skeletal morphology during maturation.^45^ These findings underscore the dual role of estrogen as both a metabolic modulator and a skeletal regulator. Progesterone similarly contributes to adipocyte differentiation, acting synergistically with estrogen during adipogenesis. Experimental evidence demonstrates that progesterone facilitates the transition of preadipocytes to lipid-accumulating adipocytes, thereby supporting maternal and fetal energy reserves.^43^ This finding aligns with physiological demands during gestation, in which enhanced energy storage is required to support fetal development and prepare for lactation.

Testosterone exhibited opposing effects, promoting muscle accretion while limiting fat deposition. Its anabolic actions include stimulation of muscle protein synthesis and inhibition of adipocyte differentiation pathways.^43^ These mechanisms explain the observed differences in carcass composition between intact and castrated males, in which higher testosterone levels support increased lean mass and reduced adiposity.^46^ Together, these hormonal influences reveal a tightly coordinated endocrine framework that modulates growth and body composition throughout development.

Growth followed a sigmoidal pattern, with early developmental stages prioritizing organogenesis and skeletal expansion, while later phases shifted toward muscle accretion, then fat deposition. The transition from rapid skeletal growth to increased muscle mass reflects progressive attainment of physiological maturity, consistent with established allometric growth models in cattle.^45^ As animals approach the finishing phase, muscle accretion slows, and adipose deposition becomes the dominant component of weight gain. Evidence from serial harvesting studies supports the disproportionate increase in fat relative to other tissues during finishing. In steers nearing maturity, fat tissue accumulated at approximately twice the rate of empty body weight (EBW), whereas lean and bone accretion declined sharply.^47^ These results are consistent with earlier work showing that, once skeletal and muscular development plateaus, metabolic priority shifts toward lipid storage, as energy is deposited more efficiently in adipose tissue.

When discussing cattle growth, it is essential to clarify whether growth is assessed within a specific life stage or across the entire lifespan, as tissue deposition patterns and underlying controls change markedly with physiological maturity. Lifelong growth follows a sigmoidal trajectory, in which early stages emphasize organogenesis and skeletal expansion, followed by a muscle-dominant phase, and ultimately a shift toward adipose deposition as animals near maturity, in line with classical allometric growth models.^45^ Serial harvest studies demonstrate that during the finishing phase, skeletal and lean tissue accretion decline while fat becomes the primary contributor to weight gain, with adipose tissue accruing at rates disproportionate to empty body weight.^47^ This transition reflects a metabolic reprioritization toward lipid storage once structural growth plateaus. Growth-modifying interventions further illustrate stage-specific growth dynamics, as anabolic implants such as trenbolone acetate and estradiol-17β can extend the muscle-dominant growth phase by sustaining protein accretion and suppressing adipogenesis,^48^ whereas advancing age and estrogenic influences accelerate fat deposition.^43^, ^44^ Collectively, these findings indicate that “growth” cannot be treated as a uniform process; rather, its interpretation and management must be aligned with the animal's developmental stage, endocrine status, and production objective, underscoring the importance of using stage-specific references appropriately when evaluating growth biology and performance outcomes.

Implant studies further demonstrate how growth-modifying technologies interact with biological maturity. Steers implanted with trenbolone acetate and estradiol-17β exhibited altered growth coefficients, characterized by increased lean accretion and reduced relative fat deposition across serial harvest endpoints.^48^ This modulation of growth curves highlights the capacity of exogenous hormones to extend the period of efficient tissue deposition by suppressing adipogenesis and sustaining protein accretion. Similarly, Wesley et al.^47^ reported that implanted steers maintained higher muscle proportions relative to carcass weight across growth stages, consistent with enhanced anabolic activity.

Taken together, these findings reveal that endocrine regulation and physiological maturity jointly determine the trajectory and efficiency of tissue deposition. Estrogen and progesterone favor adipogenic pathways, while testosterone and growth implants stimulate lean tissue growth, resulting in sex- and age-dependent variations in carcass composition. As cattle progress toward maturity, declining rates of bone and muscle accretion shift energy partitioning toward adipose tissue, aligning with observed increases in intramuscular and subcutaneous fat accumulation during finishing.^44^

Understanding these interactions is essential for optimizing production strategies. Management practices that align hormonal status, age, and nutritional inputs can effectively guide tissue deposition toward desired carcass outcomes. For example, delaying castration or using anabolic implants can prolong the muscle-growth-dominant phase, whereas early maturity or exposure to estrogenic compounds may accelerate fat deposition. These insights collectively support the development of targeted growth programs to enhance efficiency and meat quality in modern beef production systems.

### 3.3 Genetic selection, sex, and breed differences

Comparative evaluations of British and Continental breeds demonstrate substantial genetic divergence in muscling, fat deposition, and carcass outcomes. Long-term Meat Animal Research Center (MARC) datasets consistently show that Continental breeds such as Charolais, Limousin, and Simmental exhibit greater muscularity and reduced fatness than British breeds at equivalent days on feed. These phenotypic differences reflect underlying variation in muscle fiber characteristics and growth potential, with Continental breeds exhibiting larger fiber cross-sectional areas and higher proportions of glycolytic fibers, traits linked to enhanced lean deposition.^49^ In contrast, British breeds, particularly Angus, display superior marbling capacity, consistent with industry observations of enhanced intramuscular adipogenesis and associated carcass-quality advantages.

Over the past five decades, marked improvements in carcass weights further illustrate the combined effects of genetic progress and evolving management practices. Data from the United States Department of Agriculture indicate an approximate 114-kg increase in average carcass weight from the 1970s to 2023, representing a substantial shift in national production efficiency.^50^ These changes have been driven partly by sustained selection pressure on growth-related traits, as evidenced by across-breed expected progeny differences (EPDs) showing steady gains in mature size, growth rate, and muscling.^51^ Concurrent advancements in nutrition, feedlot management, and the adoption of growth-enhancing technologies (GET) have also facilitated greater weight accumulation during finishing phases.^52^ Collectively, these trends underscore the synergistic role of genetics and management in shaping modern carcass phenotypes.

Recent studies evaluating days on feed (DOF) and implant regimens further supports the contribution of management practices on carcass outcomes. Steers subjected to extended DOF and growth-implant protocols exhibited increased hot carcass weight, enhanced muscling, and improved yield metrics.^53^, ^54^ These findings align with industry-wide trends showing that the strategic use of GET and prolonged finishing feed intake amplify the expression of genetically enhanced growth potential.

Sex class exerted a significant effect on the trajectory and composition of tissue accretion. Heifers, which physiologically mature earlier than bulls and steers, displayed accelerated fat deposition, reflecting the influence of estrogen on adipogenesis and earlier attainment of physiological maturity. This pattern is consistent with established growth curves in which earlier-maturing animals shift energy partitioning toward adipose deposition sooner in the production cycle. Bulls demonstrated the greatest capacity for lean tissue deposition, driven largely by endogenous testosterone, which enhances muscle protein synthesis and inhibits adipocyte differentiation. These endocrine effects contribute to the characteristically higher muscle mass and lower fat content observed in intact males. Steers, lacking the anabolic stimulus of testosterone but maturing later than heifers, exhibited intermediate performance in both muscling and fat accumulation.

These sex-based patterns have practical implications for carcass grading and marketing. Earlier-maturing heifers tend to achieve marbling benchmarks sooner but may accumulate excessive subcutaneous fat with longer DOF, whereas bulls maximize lean gain efficiency but typically produce carcasses with lower marbling and altered fat distribution. Steers remain the predominant class in commercial feedlots due to their balanced expression of growth potential and carcass quality traits, especially under production systems that rely on implant programs and extended finishing periods.^53^, ^54^

Table 1 shows the biological types of cattle, weaning weight, and carcass composition. The combined evaluation of breed type, sex class, and modern production strategies emphasizes that carcass performance results from the interaction of genetic predisposition, endocrine influences, and environmental management. Continental breeds exhibit superior lean growth capacity, but British breeds remain advantageous for marbling-driven quality grades. Sex hormones modulate these genetically defined growth patterns, resulting in distinct deposition trajectories for heifers, steers, and bulls. Continuous genetic improvement, coupled with optimized DOF and implant technologies, has produced progressively heavier and more muscular carcasses across the U.S. beef industry, as reflected in national records from the 1970s to 2023.^50^, ^52^ These findings highlight the importance of integrated management strategies that account for biological constraints and production goals. Aligning breed selection, sex class management, nutritional strategies, and GET protocols enables producers to strategically optimize both yield and quality outcomes in contemporary beef systems.

**Table 1:** Biological types of cattle weaning weight and carcass composition.^55^, ^56^

Sire Breed	Carcass Weight (kg)	Marbling Score	Fat Thickness (mm)
Angus	289	49	16.0
Hereford	289	46	16.9
Limousine	299	39	10.8
Charolais	314	46	10.8
Simmental	305	44	11.0
Angus (2023)	422	63	16.7
Hereford (2023)	401	53	14.7
Limousine (2023)	407	53	12.1
Charolais (2023)	418	53	11.4
Simmental (2023)	418	55	12.2
All (1970–1972 Avg.)	299	45	13.0
All (2023 Avg.)	413	55	13.4

### 3.4 Nutrition and backgrounding

Across the historical and contemporary literature, backgrounding (i.e., early-life energy restriction or moderated energy intake before feedlot finishing) consistently alters the growth trajectory of beef cattle by prioritizing frame and skeletal development during the growing phase and shifting a greater proportion of nutrient partitioning toward compensatory or accelerated growth during the subsequent finishing phase. Early foundational observations on relative growth demonstrated that tissue deposition is not uniform over time and that the nutritional plane can redirect growth toward structural development rather than early fattening, thereby affecting later performance outcomes.^57^ Additionally, evidence that carcass composition and growth responses are sensitive to intake level and biological type further supports the concept that nutrition-mediated shifts in growth are both expected and measurable at the carcass level.^58^

In modern production systems, backgrounding programs are widely employed as a strategic “growth management” tool to manage feed resources and optimize subsequent finishing performance.^59^ Controlled evaluations demonstrate that manipulating the rate of gain during backgrounding leads to predictable differences in subsequent feedlot growth and carcass traits, indicating that pre-feedlot nutritional management significantly influences downstream efficiency and value.^60^ In addition, winter growing programs that vary energy density and growth rate during the growing period have been reported to affect subsequent finishing performance and body composition, reinforcing the idea that early nutritional regimes can alter later energy requirements and tissue deposition patterns.^61^

Comparisons between calf-fed and yearling (backgrounded) systems further demonstrate substantial differences in feedlot days and carcass outcomes. Studies comparing long-yearling systems with calf-fed systems report meaningful differences in feedlot duration and economics, implying that growth accrued outside the feedlot can translate to fewer finishing days and distinct carcass endpoints.^62^ Similarly, sorting steers into calf-fed, summer yearling, or fall yearling systems yields divergent feedlot durations and carcass results, illustrating that the timing and intensity of growth phases are major drivers of final carcass weight and system efficiency.^63^

Addressing the apparent contradiction between recommendations for “personalized nutrition” and the realities of group-fed beef production requires reframing personalization as biologically informed, cohort-based management rather than individual-animal feeding. Extensive evidence from backgrounding and growing-phase studies demonstrates that cattle respond predictably to differences in nutritional plane, growth rate, and timing of energy restriction or provision, with measurable downstream effects on finishing performance and carcass traits.^60^, ^61^ In practice, this enables producers to implement personalized nutrition by sorting cattle into groups based on weight, age, sex, growth system (e.g., calf-fed vs. yearling), or expected maturity pattern, as is already common in backgrounding and system-level comparisons.^62^, ^63^ Such grouping aligns nutrient supply with shared physiological stage and growth potential, effectively tailoring nutrition at the pen or cohort level. Moreover, recognition that growth capacity is influenced by earlier developmental factors, including prenatal muscle development and environmental constraints such as drought, further supports the need for flexible, stage-specific nutritional strategies that account for starting biological variation without requiring individual feeding.^64^, ^65^ Thus, personalized nutrition in beef systems is most realistically achieved through strategic sorting, phase-feeding, and system-specific ration design, allowing nutrition to be matched to biological need within herds while remaining compatible with large-scale, group-based management.

Finally, although the primary focus here is backgrounding and finishing, a broader biological context indicates that earlier developmental conditions also shape growth potential. Research documenting fetal skeletal muscle development as a function of maternal nutrition, fetal sex, and gestational age indicates that prenatal factors can constrain or enhance postnatal growth capacity, which may interact with backgrounding responses later in life.^64^ Moreover, environmental constraints, such as drought, can influence productivity and efficiency at the cow-calf level, potentially affecting the starting point for backgrounding and finishing programs.^65^

### 3.5 Composition of gain and carcass transfer

The reviewed evidence indicates that as days on feed (DOF) increase, the composition of gain progressively shifts toward a greater proportion of adipose tissue, with concomitant impacts on live performance metrics and carcass outcomes. Classic work relating intake level to carcass composition provides a mechanistic basis for this pattern: as animals approach physiological maturity, additional energy intake increasingly supports fat accretion rather than lean tissue growth.^58^ This principle aligns with subsequent research demonstrating that growth manipulation can be used to target carcass quality, implying that the timing and level of nutrition alter the lean-to-fat trajectory.^66^

Serial slaughter/serial harvest literature further quantifies a critical late-finishing phenomenon: the proportion of live weight gain retained as carcass gain, i.e., carcass transfer, increases with extended feeding. Large-pen serial slaughter syntheses report that carcass transfer may reach up to ~89% of live weight gain late in the feeding period, reflecting increasing deposition of carcass tissues (particularly fat) relative to non-carcass components as DOF increases.^67^ More recent work evaluating extended DOF confirms that performance and carcass characteristics change predictably over time, providing contemporary evidence that the marginal response to additional DOF differs from that observed in early finishing phases.^68^

Concurrently, growth-enhancing technologies (GET) are reported to increase carcass lean yield and dressing percentage, thereby modifying nutrient partitioning between carcass and non-carcass components. Serial harvest evaluations show that anabolic implants (e.g., trenbolone acetate and estradiol-17β) influence yields of carcass and non-carcass components across endpoints, indicating technology-mediated shifts in tissue accretion and distribution.^48^ Studies in calf-fed Holstein steers further document that β-adrenergic agonist use (e.g., zilpaterol hydrochloride) affects byproduct yields across serial harvest points, consistent with altered partitioning toward saleable carcass tissues.^69^ Randomized and multisite analyses in heifers also show that implant programs interact with DOF to influence feedlot performance and carcass traits, supporting the view that both time on feed and technology shape carcass outcomes.^70^ Additionally, DOF effects on performance and carcass characteristics in crossbred beef × dairy heifers corroborate the notion that time-dependent changes are not unique to a single biological type, though the magnitude may vary by genotype and sex class.^71^

## 4 Environmental and management factors

The growth physiology of cattle is influenced significantly by a complex interplay of environmental and management factors. A series of studies have focused on understanding how these elements affect cattle performance, particularly in the face of challenges such as climate change and varying farming practices. This discussion synthesizes recent findings on these factors, highlighting key environmental stressors, genetic influences, and management strategies that can enhance cattle growth and productivity. One of the most pressing environmental factors affecting cattle growth is heat stress, particularly in tropical and subtropical regions. Studies have shown that elevated temperatures negatively impact growth rates, milk production, and overall cattle welfare. For instance, it is reported that when ambient temperatures exceed 25 °C, the physiological stress on cattle can lead to significant declines in performance, as they struggle to maintain metabolic homeostasis and thermoregulation.^72^, ^73^ Becker et al.^72^ further note that the increasing average temperatures expected due to climate change exacerbate these challenges, emphasizing the need for effective heat-stress management strategies.

To illustrate, in studies involving Friesian Holstein cattle in Indonesia, heat stress was associated with decreased milk yields and reproductive performance, demonstrating the detrimental effects of the tropical environment on cattle originally bred for temperate climates.^74^ Similarly, Braz et al.^75^ and Neto et al.^76^ discuss genotype-by-environment interactions that can account for variability in growth traits such as weaning and yearling weights, revealing that both genetics and environment interact significantly with growth outcomes. Besides heat, other environmental stressors, such as noise pollution, also affect cattle growth physiology. Research indicates that chronic noise exposure can lead to stress responses that affect both behavioral and physiological parameters in cattle, potentially reducing growth performance.^77^, ^78^ Moreover, Santana reported that climate variability, including humidity and precipitation, can influence fodder quality and availability, thereby further affecting growth performance and health outcomes in cattle.^73^, ^79^ Understanding these multifaceted environmental influences is crucial for devising effective growth management strategies.

Nutritional management is a critical determinant of cattle growth and overall health. Optimizing feed efficiency through tailored nutrition can significantly enhance growth trajectories in cattle. As highlighted by Nikkhah and Alimirzaei,^80^ well-managed feeding practices, including the use of direct-fed microbials, can improve gut health and increase growth rates in calves, ultimately enhancing productivity. Furthermore, Santana^79^ emphasizes the importance of integrating environmental data with nutritional practices to improve the efficiency of genetic evaluations for pasture-raised cattle. By doing so, managers can align feeding strategies with the specific environmental conditions, thereby supporting better growth outcomes.

The management of cattle prior to slaughter also plays a pivotal role in determining meat quality and overall animal welfare. Davis et al.^81^ point out that factors such as transportation, environmental conditions, and handling procedures can significantly influence the welfare and physiological state of cattle, thereby affecting post-harvest meat quality. High-stress pre-slaughter environments have been linked to poorer meat quality and reduced economic returns, indicating the necessity for refined management protocols during this critical period of the production cycle. Genetic selection, guided by an understanding of environmental impacts, is essential for enhancing growth traits in cattle. Research suggests that including genotype-by-environment interaction data in breeding programs can yield more resilient cattle suited to specific environments, thereby improving growth performance and adaptive capacity.^76^, ^82^ Identifying and selecting for traits that confer advantages under specific environmental conditions can enhance the effectiveness and sustainability of breeding programs.

Significant advances have been made in understanding the environmental and management factors that affect growth physiology in cattle. Greater awareness of the challenges posed by climate change, nutritional management, and pre-slaughter conditions forms the basis for developing more effective management strategies. The integrated approach, taking into account both genetic makeup and environmental needs, will be key to enhancing the productivity and welfare of cattle, ultimately ensuring the sustainability of beef and dairy production systems globally. This holistic view promotes continuous improvement in cattle management practices across an ever-changing environmental landscape, paving the way for innovations in livestock farming.

## 5 Future research and recommendations

The growth physiology of cattle remains a vital area of research that intersects with genetics, nutrition, and disease management. Advancements in technology and methodologies present opportunities to deepen our understanding of this complex field. This synthesis discusses potential future research directions and practical recommendations based on recent literature and emerging trends. Research into genetic markers and epigenetic influences on growth traits is essential. Previous studies have shown the importance of quantitative trait loci (QTL) for traits such as growth and body composition in cattle.^83^ Future studies should aim to elucidate the mechanisms by which these genetic factors influence growth and efficiency. Furthermore, exploring epigenetic modifications, as outlined by Feeney et al., can provide insights into how environmental factors affect growth traits and could ultimately lead to targeted genetic improvements in cattle.^84^

The integration of precision nutrition into cattle production is gaining momentum. The use of telemetric systems for continuous monitoring of ruminal functions illustrates the potential for real-time data to optimize nutritional strategies.^85^ Future research should focus on developing personalized nutrition plans that account for individual animal health, genetics, and environmental factors, thereby enhancing growth performance and feed efficiency.^86^ Adopting new technologies such as virtual fencing could revolutionize cattle management practices by enabling dynamic feeding strategies that align with pasture availability and animal behavior.^87^ Research should evaluate the long-term impacts of such technologies on animal welfare and growth rates to determine their efficacy in different farming systems.

Cattle are often susceptible to various infectious diseases, which have direct implications for growth and productivity. Future studies should investigate the relationships between disease resistance genes and growth metrics. For instance, understanding the role of microRNAs in immune responses can illuminate pathways by which cattle resist infections such as Neospora caninum, thereby impacting growth outcomes.^88^, ^89^ Focused research on enhancing immune responses through genetic selection could lead to healthier cattle with improved growth rates.

As attention to animal welfare increases, the development of methods to monitor physiological health metrics is crucial. Studies comparing various sampling techniques for monitoring volatile organic compounds can help refine health assessments and identify animals at risk for growth impairments due to illness.^90^ Future research should compare these methodologies across diverse settings to establish standardized health monitoring protocols.

Research into resource allocation within cattle production systems could yield strategies to enhance growth efficiency. Building upon existing models that examine resource acquisition and allocation will help identify the most effective ways to partition available nutrients for optimal growth.^86^ These insights could drive improvements in feed formulation and animal management practices, fostering sustainable production methods.

As climate variability changes the agricultural landscape, research into cattle adaptability to adverse conditions is vital. Investigating the physiological adaptations of cattle breeds to various environmental stresses could provide data to select for traits that enhance resilience and growth performance.^91^ Future studies should also incorporate how nutritional strategies can mitigate the impacts of climate-induced stressors on cattle growth.

Future research on growth physiology in cattle should encompass integrative studies of genetics, innovative nutritional strategies, improved health monitoring, and sustainable production practices. Enhancing our understanding of these interconnected areas will help drive improvements in cattle growth efficiency while addressing the pressing challenges of livestock production sustainability and animal welfare. Stakeholders in the cattle industry, including researchers, farmers, and policymakers, should collaborate effectively to align research priorities with practical applications that meet the demands of the evolving agricultural landscape.

## 6 Conclusion

This bibliometric and integrative physiological synthesis reveals that research on cattle growth has evolved from traditional assessments of tissue development toward a multidimensional framework that incorporates genetics, nutrition, environmental stressors, and sustainability-driven priorities. The findings show that prenatal programming establishes the fundamental blueprint for muscle, adipose, and skeletal development, while postnatal growth is shaped by hormonal regulation, nutrient intake, and physiological maturity. Contemporary studies increasingly emphasize the role of genetic selection, particularly of traits such as residual feed intake, alongside sex- and breed-related variation that determines patterns of lean and fat deposition. Nutrition and backgrounding strategies further modulate these intrinsic biological pathways, indicating that growth trajectories are highly responsive to early-life feeding regimes and management conditions.

The bibliometric results underscore a rapidly expanding scientific interest, with notable growth in keywords including feed efficiency, metabolomics, and proteomics. These emerging research clusters highlight a shift toward understanding the molecular and metabolic networks that regulate growth, nutrient partitioning, stress resilience, and overall production efficiency. Environmental and management factors, especially heat stress, climate variability, and pre-slaughter handling, were identified as increasingly critical determinants of growth outcomes. Collectively, these insights demonstrate that sustainable cattle production requires integrating physiological knowledge with precision nutrition, genetic adaptation, and environmental management.

The main implication of this study is that improving the sustainability of cattle growth cannot rely on any single domain of research; rather, it demands an integrated approach that links developmental physiology with innovative tools such as omics technologies, advanced genetic evaluations, and climate-adapted management practices. By mapping global research trends, this study contributes to the existing body of knowledge by clarifying the field's conceptual structure, identifying emerging scientific priorities, and revealing geographic imbalances that may influence knowledge development.

Future research should further explore multi-omics integration, gene–environment interactions, precision feeding technologies, and physiological biomarkers that predict resilience under climate stress. Additionally, there is a need for more region-specific studies in underrepresented countries to expand the global relevance of growth physiology research. Overall, this work demonstrates that advancing sustainable cattle production requires coordinated efforts across genetics, nutrition, physiology, and environmental sciences, offering a roadmap for future inquiry and innovation.

## CRediT authorship contribution statement

Ni Made Ayu Gemuh Rasa Astiti*: Writing – original draft, Software, Resources, Project administration, Methodology, Investigation, Conceptualization. **I Gusti Ayu Dewi Seri Rejeki:** Writing – review & editing, Resources, Methodology, Investigation, Data curation. **Ahmad Fudholi:** Writing – review & editing, Supervision, Project administration, Methodology, Investigation.

## Declaration of competing interest

The authors declare that they have no known competing financial interests or personal relationships that could have appeared to influence the work reported in this paper.
